# The clinicopathological features of ganglioglioma with CD34 expression and BRAF mutation in patients with epilepsy

**DOI:** 10.3389/fnmol.2023.1022364

**Published:** 2023-02-23

**Authors:** Ming-Guo Xie, Xiong-Fei Wang, Jiao Qiao, Jian Zhou, Yu-Guang Guan, Tian-Fu Li, Xue-Ling Qi, Guo-Ming Luan

**Affiliations:** ^1^Department of Neurosurgery, Epilepsy Center, Sanbo Brain Hospital, Capital Medical University, Beijing, China; ^2^Beijing Key Laboratory of Epilepsy, Sanbo Brain Hospital, Capital Medical University, Beijing, China; ^3^Beijing Institute for Brain Disorders, Capital Medical University, Beijing, China; ^4^Department of Neurology, Epilepsy Center, Sanbo Brain Hospital, Capital Medical University, Beijing, China; ^5^Department of Neuropathology, Epilepsy Center, Sanbo Brain Hospital, Capital Medical University, Beijing, China

**Keywords:** epilepsy, Ganglioglioma, CD34, BRAF, surgery

## Abstract

**Objective:**

The aim of the study was to evaluate the clinicopathological features, as well as the surgical prognosis, of epilepsy-associated gangliogliomas (GG) with CD34 expression and BRAF^V600E^ mutation.

**Methods:**

Clinical data of patients who underwent epilepsy surgery for GG were retrospectively studied. Univariate and multivariate analyses were performed to evaluate the correlations of clinical and pathological factors with molecular markers of CD34 expression and BRAF^V600E^ mutation in GG.

**Results:**

A total of 208 patients with GG had immunohistochemical detection of CD34 expression (positive/negative: 184/24), and among them, 89 patients had immunohistochemical detection of BRAF^V600E^ mutation (positive/negative: 54/35). By univariate and multivariate analyses, seizure aura (*p* = 0.025), concordance of ictal electroencephalogram (EEG) findings (*p* = 0.045) and medial temporal tumor (*p* = 0.030) were found to be related to CD34 expression, but only hospitalization time (*p* = 0.042) was different for BRAF-mutated status. In addition, drug-resistant epilepsy (*p* = 0.040) and concordance of interictal EEG findings (*p* = 0.009) were found to be associated with tumor progression-free survival (PFS) in univariate analysis, but only concordance of interictal EEG findings was with significance in multivariate analysis. However, CD34 expression or BRAF^V600E^ mutation in GG was not found to be associated with surgical outcomes of seizure control and tumor PFS.

**Conclusion:**

The CD34 expression or BRAF^V600E^ mutation in GG may partly influence the distribution of clinicopathological features of patients with epilepsy, but they may be not able to predict the surgical prognosis of seizure outcome and tumor recurrence.

## Introduction

Brain tumors are frequently met in patients with epilepsy surgery, and among them, gangliogliomas (GG) are the most common tumor entities (Blumcke et al., 2017; Slegers and Blumcke, 2020). Recently, the molecular markers of CD34 and BRAF mutation are interestingly found to be associated with brain tumors with epilepsy, especially with epilepsy-associated GG (Blümcke et al., 1999; Deb et al., 2006; Schindler et al., 2011; Giulioni et al., 2019; Xing et al., 2021).

CD34 is a transmembrane phosphoglycoprotein that was first identified on hematopoietic progenitor cells and subsequently vascular endothelial progenitors, multipotent mesenchymal stromal cells, and epithelial progenitors, etc., thus being regarded as a general marker of progenitor cells (Guo et al., 2003; Sidney et al., 2014). CD34 is transiently expressed in the central nervous system during early neurulation and cannot be detected in mature neuroectodermal cell progenies in the normal brain (Blümcke et al., 1999). Although CD34-positive cells have been reported in gliosarcoma and giant cell variant of glioblastoma, or non-neoplastic glioneuronal hamartias or hamartomas, they are particularly represented in low-grade or developmental brain tumors, such as GG, pleomorphic xanthoastrocytoma (PXA), dysembroplastic neuroepithelial tumor (DNT) and pilocytic astrocytoma (PA), all of which are quite associated with chronic epilepsy (Blümcke et al., 1999; Reifenberger et al., 2003; Deb et al., 2006; Giulioni et al., 2019).

BRAF (v-raf murine sarcoma viral oncogene homolog B1) is a member of the RAF family of serine/threonine protein kinases, playing a critical role in transducing signals from membrane-bound, GTP-loaded RAS proteins to MEK and ERK kinases (RAS/RAF/MEK/ERK pathway; Dougherty et al., 2010; Drosten and Barbacid, 2020). The vast majority of BRAF mutations (>90%) affect a mutational hot spot at amino acid position 600 and are characterized by the exchange of Valine by Glutamate, thus referred to as BRAF Val600Glu (or BRAF^V600E^), which generates a constitutively active monomeric protein with high kinase activity that does not require RAS signaling (Davies et al., 2002; Dougherty et al., 2010). BRAF is mutated in about 8% of all human cancers, and these mutations primarily occur in melanomas and at much lower frequency in thyroid, lung, and colorectal cancer (Davies et al., 2002; Drosten and Barbacid, 2020). Recently, BRAF^V600E^ mutations are found in brain tumors, mainly affecting glial or glioneuronal tumors, such as PXA, GG, DNT, and PA, as well as pediatric astrocytoma (Davies et al., 2002; Schindler et al., 2011; Xing et al., 2021), but glioblastoma and other gliomas were with low frequency or absence of mutations, and none of them in non-glial tumors (Schindler et al., 2011; Xing et al., 2021). More recently, the monoclonal BRAF^V600E^ mutation-specific antibody *via* immunohistochemistry (IHC) detection has been found and widely used to screen for BRAF^V600E^ mutation in the diagnostic work-up (Blümcke et al., 2016; Slegers and Blumcke, 2020), since the first BRAF^V600E^ specific antibody was reported in 2011 (clone VE1; Capper et al., 2011).

Although the molecular expression of CD34 and BRAF^V600E^ mutation could frequently and exclusively occur in GG with epilepsy, their clinical and pathological features were not yet well defined, as well as the prediction of long-term seizure outcome and tumor recurrence or progression (Blümcke et al., 1999; Dahiya et al., 2013; Zhang et al., 2017; Giulioni et al., 2019; Xing et al., 2021). Thus, we aimed to evaluate the associations of clinicopathological features, as well as surgical prognosis, with molecular expression of CD34 and BRAF^V600E^ mutations in GG with epilepsy.

## Materials and methods

### Patient selection

A retrospective chart review was conducted for all patients with epilepsy who underwent surgical treatment for GG between 2008 and 2021 at Sanbo Brain Hospital, Capital Medical University. This study was approved by the Capital Medical University Sanbo Brain Hospital Ethics Committee.

Patients who had epilepsy caused by brain tumors that were histopathologically confirmed as low-grade GG were enrolled in the study. The clinical data of patients with tumors that were detected by IHC with molecular markers of CD34 and BRAF^V600E^ mutation was analyzed. Finally, a total of 208 consecutive patients who had tumors with IHC examination of CD34 expression, including 89 patients with IHC examination of BRAF^V600E^ mutation, were enrolled in the study.

### Preoperative evaluation and surgery

All patients underwent an individualized preoperative evaluation, including detailed medical history and physical examination, seizure semiology, video electroencephalogram (EEG) and brain magnetic resonance imaging (MRI). The lesion size was represented by the mean tumor diameter of T1-weighted MRI scans. The video EEG monitoring was performed in all patients for at least 16 h, and the concordant EEG findings of interictal epileptiform discharges and ictal seizure rhythms were defined as epileptiform discharge sources localized in the same tumor-invading brain hemisphere.

After detailed preoperative evaluations by neurologists, neurosurgeons, neuroradiologists and electrophysiologists, surgical plans were made. The aim of the operation was to remove the tumor and relevant epileptogenic zone (EZ). The EZ was determined by the findings of the detailed preoperative evaluation and/or intraoperative electrocorticography (ECoG). Intraoperatively, neurological electrophysiological monitoring and neuronavigation were also performed for safe tumor resection. In particular, according to the resection extent of brain tissue (determined by operative records and postoperative MRI), extensive tumor resection was defined as resection of both tumor and peritumoral cortex (or hippocampus), while simple tumor resection was those with simply resection of the tumor.

### Immunohistochemical staining and pathological diagnosis

The surgically removed brain tissue specimens were fixed with 3.7% neutral formaldehyde, embedded in conventional paraffin, sliced into 5-μm thick sections, and then stained with hematoxylin and eosin (H&E). Immunohistochemical staining was performed with the following primary antibodies: anti-BRAF^V600E^ (Spring Bioscience, monoclonal, clone VE1, 1:50), glial fibrillary acidic protein (GFAP; Dako, 1:1000), neuronal nuclear antigen (NeuN; Chemicon, 1:2000), neurofilament (NF; Zymed, 1:100), synaptophysin (Biogenics, 1:50), oligodendrocyte transcription factor 2 (Olig-2; Immuno-Biological Laboratories, 1:500), epithelial cell membrane antigen (EMA; Zymed, 1:100), Ki-67 (MIB-1; OriGene, monoclonal, clone UMAB107, 1:200), p53 (Zymed, 1:100), CD68 (Bio-Rad, 1:500), CD34 (Zymed, monoclonal, clone QBEnd 10, 1:50), isocitrate dehydrogenase (IDH1/2; Zymed, 1:1000).

Histopathological records were systematically reviewed by two experienced neuropathologists according to the WHO classification scheme from 2016, including a panel of immunohistochemical markers. Ki-67 proliferation index was defined by the percentage of Ki-67 positive cells in the total cell population at 40 magnifications for 10 microscopic fields. In particular, tumors with IHC detection of CD34 expression and BRAF^V600E^ mutation were analyzed in terms of their associations with clinical and pathological features.

### Follow-up examination

Patients were periodically followed up at the 3rd month and 6th month postoperatively and yearly thereafter. Follow-up evaluations of seizure and tumor recurrence or progression, as well as neurological statuses, were performed by neurosurgeons at the clinic and/or by telephone interview in each patient. Favorable seizure outcomes with Engel classification were defined as Engel class I, and unfavorable seizure outcomes were Engel class II-IV at the last follow-up evaluation.

### Study variables and statistical analysis

Clinical variables of interest were evaluated between two groups of patients with or without CD34 expression and BRAF^V600E^ mutation, including patient or demographic characteristics, tumor or pathological characteristics, seizure semiology and electrophysiological findings, surgical and follow-up variables.

Continuous variables were described with medians and interquartile ranges (IQR), while categorical variables were described with absolute and relative (%) frequencies. Descriptive statistics between compared groups were analyzed by *t* tests and *χ*^2^ tests for continuous and categorical variables, respectively. When necessary, Fisher’s exact test and the Kruskal-Wallis rank-sum test were used. Variables showing a *p* < 0.05 in the univariate analysis were then entered into the multivariate binary logistic or Cox regression model. Statistical tests were considered significant if *p* < 0.05. Odds ratios (OR) and hazard ratios (HR) were presented with 95% confidence intervals (CI). All data were analyzed using the software package SPSS, version 21.

## Results

### Patient demographics

Of the 208 patients, 76 patients (36.5%) were female and 89 patients (42.8%) were children (age < 18 years old). The median age at surgery was 20 years (IQR: 11–26 years), the median age of seizure onset was 10 years (IQR: 4–17 years), and the median duration of epilepsy was 60 months (IQR: 18–144 months; Table 1). Upon admission, 163 patients (78.4%) were with drug-resistant epilepsy.

**Table 1:** Univariate analysis of the relationships between CD34 expression in GG and clinicopathology in 208 patients.

Variable	Subtype	CD34 expression		In total	*p* value
		CD34 (−)	CD34 (+)		
BRAF^V600E^ mutation, *n* (%)	*Braf* (−)	2 (5.7%)	33 (94.3%)	35	0.069
	Braf (+)	3 (5.6%)	51 (94.4%)	54	
	Unknown^a^	19 (16%)	100 (84%)	119	
Patient gender, *n* (%)	Male	16 (12.1%)	116 (87.9%)	132	0.729
	Female	8 (10.5%)	68 (89.5%)	76	
Patient population, *n* (%)	Children	14 (15.7%)	75 (84.3%)	89	0.102
	Adult	10 (8.4%)	109 (91.6%)	119	
Drug-resistant epilepsy, *n* (%)	No	5 (11.1%)	40 (88.9%)	45	0.919
	Yes	19 (11.7%)	144 (88.3%)	163	
Seizure type, *n* (%)	Focal	16 (11.5%)	123 (88.5%)	139	0.986
	Generalized	8 (11.6%)	61 (88.4%)	69	
Seizure aura, *n* (%)	No	16 (16.7%)	80 (83.3%)	96	0.032^d^
	Yes	8 (7.1%)	104 (92.9%)	112	
History of GTCS, *n* (%)	No	9 (10.5%)	77 (89.5%)	86	0.684
	Yes	15 (12.3%)	107 (87.7%)	122	
History of SE, *n* (%)	No	24 (11.8%)	179 (88.2%)	203	0.913
	Yes	0 (0%)	5 (100%)	5	
Seizure frequency, *n* (%)	Daily	12 (18.2%)	54 (81.8%)	66	0.172
	Weekly	5 (6.3%)	74 (93.7%)	79	
	Monthly	5 (11.6%)	38 (88.4%)	43	
	Yearly	2 (10%)	18 (90%)	20	
Interictal EEG findings, *n* (%)	Discordant	6 (14%)	37 (86%)	43	0.851
	Concordant	16 (10.8%)	132 (89.2%)	148	
	Unknown^b^	2 (11.8%)	15 (88.2%)	17	
Ictal EEG findings, *n* (%)	Discordant	9 (20.9%)	34 (79.1%)	43	0.024^d^
	Concordant	8 (7.5%)	99 (92.5%)	107	
	Unknown^b^	7 (12.1%)	51 (87.9%)	58	
Resection extent, *n* (%)	Simple tumor resection	11 (14.1%)	67 (85.9%)	78	0.370
	Extensive tumor resection	13 (10%)	117 (90%)	130	
Tumor type, *n* (%)	GG	24 (12.1%)	174 (87.9%)	198	0.507
	GG-nos	0 (0%)	10 (100%)	10	
Tumor side, *n* (%)	Right	11 (10.2%)	97 (89.8%)	108	0.526
	Left	13 (13%)	87 (87%)	100	
Tumor location, *n* (%)	Temporal	14 (9.5%)	133 (90.5%)	147	0.359
	Non-temporal	7 (17.1%)	34 (82.9%)	41	
	Multilobe	3 (15%)	17 (85%)	20	
Medial temporal tumor, *n* (%)	No	12 (8.4%)	131 (91.6%)	143	0.035^d^
	Yes	12 (18.5%)	53 (81.5%)	65	
Tumor calcification, *n* (%)	No	14 (10.4%)	121 (89.6%)	135	0.473
	Yes	10 (13.7%)	63 (86.3%)	73	
Tumor encystation, *n* (%)	No	21 (11.9%)	155 (88.1%)	176	0.908
	Yes	3 (9.4%)	29 (90.6%)	32	
Ki67 index, *n* (%)	0–1%	16 (11.2%)	127 (88.8%)	143	0.815
	2-5%^c^	8 (12.3%)	57 (87.7%)	65	
Tumor-associated FCD, *n* (%)	No	14 (9.4%)	135 (90.6%)	149	0.124
	Yes	10 (16.9%)	49 (83.1%)	59	
Concomitant HS, *n* (%)	No	22 (11.6%)	168 (88.4%)	190	1.000
	Yes	2 (11.1%)	16 (88.9%)	18	
Seizure outcome, *n* (%)	Engel I	18 (10.9%)	147 (89.1%)	165	0.791
	Engel II-IV	4 (14.8%)	23 (85.2%)	27	
Tumor recurrence, *n* (%)	No	24 (11.8%)	179 (88.2%)	203	0.913
	Yes	0 (0%)	5 (100%)	5	
Age at surgery, median (IQR)	In years	10.5 (3–24.7)	20 (12.5–26)	20 (10.6–25.7)	0.060
Age of seizure onset, median (IQR)	In years	4.5 (1–12)	10.25 (4.1–16.9)	10 (4–16.4)	0.017^d^
Duration of epilepsy, median (IQR)	In months	42 (12.3–129)	60 (18–153)	60 (18–144)	0.476
Tumor size, median (IQR)	In millimeter	20 (15.5–19)	17.5 (15–20)	17.5 (15–20)	0.258
Hospitalization time, median (IQR)	In days	27 (21–32)	25 (19–30.7)	25 (19.3–31)	0.169
Follow-up time, median (IQR)	In months	72.5 (30.7–99.7)	52 (25–76)	54 (25–78.7)	0.031^d^

GG, ganglioglioma; GG-nos, ganglioglioma, not otherwise specified; GTCS, generalized tonic–clonic seizure; SE, status epilepticus; EEG, electroencephalogram; FCD, focal cortical dysplasia; HS, hippocampus sclerosis; IQR, interquartile range. ^a^The unknown cases were those without immunohistochemical examination of BRAF^V600E^ mutation. ^b^Patients with unknown results in lateral concordant EEG findings of interictal epileptiform discharges and of ictal seizure rhythms were recorded in 17 cases (no interictal epileptiform discharges or normal EEG findings) and 58 cases (no ictus during video EEG monitoring), respectively. ^c^Including 2 cases with ki67 index recorded at 8 and 12%, respectively. ^d^*p* < 0.05, with significance.

### Tumor characteristics

Of the 208 tumors found by MRI, 100 cases (48.1%) were in the left brain. In particular, 147 patients (70.7%) had tumors located in the temporal lobe. Tumors located in the frontal, parietal, occipital, insular and multiple lobes were found in 17 (8.2%), 11 (5.3%), 10 (4.8%), 3 (1.4%), and 20 (9.6%) cases, respectively. The median tumor size was 17.5 mm (IQR: 15–20 mm; Table 1).

According to postoperative pathological records of surgical specimens, all 208 lesions were diagnosed as low-grade GG (WHO grade I/174 or II/34), including GG with mixed characteristics of DNT (Xing et al., 2021), PXA (Giulioni et al., 2019) and astrocytoma (Slegers and Blumcke, 2020) in 10 cases (4.8%). Tumor-associated focal cortical dysplasia (FCD) was recorded in 59 patients (28.4%), including 23 cases (11.1%) of FCD II. Concomitant hippocampus sclerosis (HS) was found in 18 patients (8.7%). Tumors with tissue calcification and encystation were recorded in 73 cases (35.1%) and 32 cases (15.4%), respectively. The Ki67 index of tumor tissue was categorized into three subgroups: 0–1% (143 cases), 2–5% (63 cases), and 6–12% (2 cases). In particular, the IHC detection of CD34 positive expression was found in 184 (88.5%) patients, while CD34 negative expression was found in 24 patients (11.5%). Of the 89 patients (42.8%) with IHC detection of BRAF^V600E^ mutation, 54 cases (60.7%) were BRAF positive (Table 1). In addition, 180 cases (86.5%) were tested with IDH mutations, but no IDH (+) was found in all tested lesions of GG.

### Seizure semiology and electrophysiological findings

Before surgery, 66 patients (31.7%) complained of daily seizure onsets, while the other 142 patients (68.3%) experienced seizure onsets weekly (79), monthly (43), quarterly or yearly (Prabowo et al., 2014). A total of 139 patients (66.8%) had focal seizures as the most common seizure onset in recent years, while 69 patients (33.2%) had generalized seizures. In addition, history of seizure auras, generalized tonic–clonic seizures (GTCS) and status epilepticus (SE) were recorded in 112 (53.8%), 122 (58.7%), and 5 (2.4%) patients, respectively.

Regarding video EEG findings, concordant interictal EEG findings were found in 148 patients (71.1%), while discordant findings were in 43 patients (20.7%); 17 patients (8.2%) were with unknown results due to lack of significant epileptiform discharges or being in a normal EEG setting. Concordant EEG findings of ictal seizure rhythms were found in 107 patients (51.4%), and discordant findings were found in 43 patients (20.7%), but 58 patients (27.9%) were with unknown results due to no significant ictal seizures (Table 1).

### Surgical results

Intraoperative ECoG monitoring was performed in 171 patients (82.2%). Complete tumor resection was achieved in 206 patients (99%), and 2 cases were with subtotal tumor resection because of tumors invading brain eloquent areas. In total, extensive tumor resection was performed in 130 patients (62.5%), and simple tumor resection was in 78 patients (37.5%).

Postoperatively, 29 patients (13.9%) had acute seizures within the first 2 weeks after surgery. Operation-associated complications were met in 27 patients (13%), including venous thrombosis (Blumcke et al., 2017), pulmonary infection (Giulioni et al., 2019), intracranial infection (Xing et al., 2021), hemorrhagic apoplexy (Blumcke et al., 2017), cerebral infarction (Giulioni et al., 2019), incision infection or poor healing (Blümcke et al., 1999), and others (7; such as electrolyte disorders, urinary tract infection and gastrointestinal dysfunction). New neurological deficits were recorded in 22 patients (10.6%), including, muscle weakness (Dougherty et al., 2010), impaired vision (Xing et al., 2021), aphasia (Giulioni et al., 2019), decreasing memory (Deb et al., 2006), mental disorder (Giulioni et al., 2019) and eyelid drooping (Blumcke et al., 2017). The median time of hospitalization was 25 days (IQR: 19–31 days; Table 1).

### Follow-up outcomes

All patients were followed up, except for 12 patients (5.8%) lost, with the median follow-up time of 54 months (IQR: 25–79 months). Of 192 patients who were followed up for at least 12 months, 165 patients (85.9%) were seizure-free and had a favorable seizure outcome (Engel class I), while 27 patients (14.1%) had an unfavorable seizure outcome (Engel class II/9, III/13 and IV/5; Table 1). In total, 146 patients (70.2%) had anti-epileptic drugs reduced (40) or discontinued (106). During the whole follow-up period, 5 (2.6%) patients had tumor recurrence, including one with subtotal tumor resection, and the accumulated 10-year tumor progression-free survival (PFS) was 96%. Among them, 3/5 of cases had seizure recurrence, and 2 cases of GG had malignant progression.

### Univariate and multivariate analyses

Clinical and pathological factors in 208 patients were compared between two groups [tumor with CD34 (+) vs. CD34 (−)] (Table 1). Significant differences were found in age of seizure onset (*p* = 0.017), seizure aura (*p* = 0.032), concordance of ictal EEG findings (*p* = 0.024) and medial temporal tumor (*p* = 0.035) by univariate analysis. In particular, surgical outcomes of seizure control (*p* = 0.791) and tumor recurrence (*p* = 0.913) were not found with differences between two groups. Multivariate binary logistic regression analysis finally included the seizure aura (*p* = 0.025, OR = 2.94), the concordance of ictal EEG findings (discordant vs. concordant; *p* = 0.045, OR = 0.35) and the medial temporal tumor (*p* = 0.030, OR = 0.37) into the predicting model of GG with CD34 positive expression (Table 2).

**Table 2:** Multivariate analysis of the relationships between CD34 expression in GG and clinicopathology in 208 patients.

Variables	*B*	OR (95% CI)	*p* value
Seizure aura, *n* (%)	1.079	2.94 (1.15–7.56)	0.025
Ictal EEG findings, *n* (%)			0.126
discordant vs. concordant	−1.081	0.34 (0.12–0.97)	0.045
discordant vs. unknown (no ictus)	−0.357	0.70 (0.23–2.10)	0.524
Medial temporal tumor, *n* (%)	−0.998	0.37 (0.15–0.91)	0.030

GG, ganglioglioma; EEG, electroencephalogram; OR, odds ratio; CI, confidence interval.

The clinical and pathological features of 89 patients who had IHC detection of BRAF^V600E^ mutation were also compared between two groups [tumor with BRAF (+) vs. BRAF (−)] (Table 3). Significant differences were found in hospitalization time (*p* = 0.042), and statistic tendency (0.05 < *p* < 0.1) was found in concordance of ictal EEG findings (concordant vs. discordant; *p* = 0.078) and age at surgery (*p* = 0.064), but none in surgical outcomes of seizure control (*p* = 0.895) and tumor recurrence (*p* = 1.000).

**Table 3:** Univariate analysis of the relationships between BRAF^V600E^ mutation in GG and clinicopathology in 89 patients.

Variable	Subtype	BRAF^V600E^ mutation		In total	*p* value
		Braf (−)	Braf (+)		
CD34 expression	CD34 (−)	2 (40%)	3 (60%)	5	0.975
	CD34 (+)	33 (39.3%)	51 (60.7%)	84	
Patient gender, *n* (%)	Male	19 (35.8%)	34 (64.2%)	53	0.415
	Female	16 (44.4%)	20 (55.6%)	36	
Patient population, *n* (%)	Children	17 (45.9%)	20 (54.1%)	37	0.281
	Adult	18 (34.6%)	34 (65.4%)	52	
Drug-resistant epilepsy, *n* (%)	No	12 (50%)	12 (50%)	24	0.210
	Yes	23 (35.4%)	42 (64.6%)	65	
Seizure type, *n* (%)	Focal	22 (40.7%)	32 (59.3%)	54	0.734
	Generalized	13 (37.1%)	22 (62.9%)	35	
Seizure aura, *n* (%)	No	14 (34.1%)	27 (65.9%)	41	0.355
	Yes	21 (43.8%)	27 (56.3%)	48	
History of GTCS, *n* (%)	No	17 (38.6%)	27 (61.4%)	44	0.895
	Yes	18 (40%)	27 (60%)	45	
History of SE, *n* (%)	No	35 (40.2%)	52 (59.8%)	87	0.675
	Yes	0 (0%)	2 (100%)	2	
Seizure frequency, *n* (%)	Daily	11 (40.7%)	16 (59.3%)	27	0.935
	Weekly	13 (37.1%)	22 (62.9%)	35	
	Monthly	8 (44.4%)	10 (55.6%)	18	
	Yearly	3 (33.3%)	6 (66.7%)	9	
Interictal EEG findings, *n* (%)	Discordant	6 (26.1%)	17 (73.9%)	23	0.296
	Concordant	26 (44.8%)	32 (55.2%)	58	
	Unknown^a^	3 (37.5%)	5 (62.5%)	8	
Ictal EEG findings, *n* (%)	Discordant	4 (21.1%)	15 (78.9%)	19	0.078^c^
	Concordant	19 (45.2%)	23 (54.8%)	42	
	Unknown^a^	12 (42.9%)	16 (57.1%)	28	
Resection extent, *n* (%)	Simple tumor resection	15 (41.7%)	21 (58.3%)	36	0.709
	Extensive tumor resection	20 (37.7%)	33 (62.3%)	53	
Tumor type, *n* (%)	GG	32 (38.6%)	51 (61.4%)	83	0.903
	GG-nos	3 (50%)	3 (50%)	6	
Tumor side, *n* (%)	Right	17 (37%)	29 (63%)	46	0.636
	Left	18 (41.9%)	25 (58.1%)	43	
Tumor location, *n* (%)	Temporal	23 (35.4%)	42 (64.6%)	65	0.456
	Non-temporal	8 (50%)	8 (50%)	16	
	Multilobe	4 (50%)	4 (50%)	8	
Medial temporal tumor, *n* (%)	No	25 (42.4%)	34 (57.6%)	59	0.409
	Yes	10 (33.3%)	20 (66.7%)	30	
Tumor calcification, *n* (%)	No	18 (34%)	35 (66%)	53	0.209
	Yes	17 (47.2%)	19 (52.8%)	36	
Tumor encystation, *n* (%)	No	27 (37%)	46 (63%)	73	0.334
	Yes	8 (50%)	8 (50%)	16	
Ki67 index, *n* (%)	0–1%	21 (39.6%)	32 (60.4%)	53	0.945
	2–5%^b^	14 (38.9%)	22 (61.1%)	36	
Tumor-associated FCD, *n* (%)	No	33 (39.8%)	50 (60.2%)	83	1.000
	Yes	2 (33.3%)	4 (66.7%)	6	
Concomitant HS, *n* (%)	No	33 (40.7%)	48 (59.3%)	81	0.624
	Yes	2 (25%)	6 (75%)	8	
Seizure outcome, *n* (%)	Engel I	28 (37.8%)	46 (62.2%)	74	0.895
	Engel II-IV	4 (40%)	6 (60%)	10	
Tumor recurrence, *n* (%)	No	35 (39.8%)	53 (60.2%)	88	1.000
	Yes	0 (0%)	1 (100%)	1	
Age at surgery, median (IQR)	In years	18 (7–24)	20.5 (14–27)	20 (10.6–25.7)	0.064^c^
Age of seizure onset, median (IQR)	In years	7 (4–18)	12 (5–18.5)	10 (4–16.4)	0.215
Duration of epilepsy, median (IQR)	In months	24 (5–120)	45 (12–186)	60 (18–144)	0.182
Tumor size, median (IQR)	In millimeter	20 (15–22)	16.3 (15.5–20)	17.5 (15–20)	0.212
Hospitalization time, median (IQR)	In days	20 (16–26)	24.5 (16.7–31.3)	25 (19.3–31)	0.042^c^
Follow-up time, median (IQR)	In months	54 (25–79)	41 (23.7–71.3)	54 (25–78.7)	0.215

GG, ganglioglioma; GG-nos, ganglioglioma, not otherwise specified; GTCS, generalized tonic–clonic seizure; SE, status epilepticus; EEG, electroencephalogram; FCD, focal cortical dysplasia; HS, hippocampus sclerosis; IQR, interquartile range. ^a^Patients with unknown results in lateral concordant EEG findings of interictal epileptiform discharges and of ictal seizure rhythms were recorded in 8 cases (no interictal epileptiform discharges or normal EEG findings) and 28 cases (no ictus during video EEG monitoring), respectively. ^b^Including 1 case with ki67 index of 12%. ^c^*p* < 0.05, with significance; 0.05 < *p* < 0.1, with statistic tendency.

### Kaplan Meier curve and Cox regression analysis

**Figure 1:**
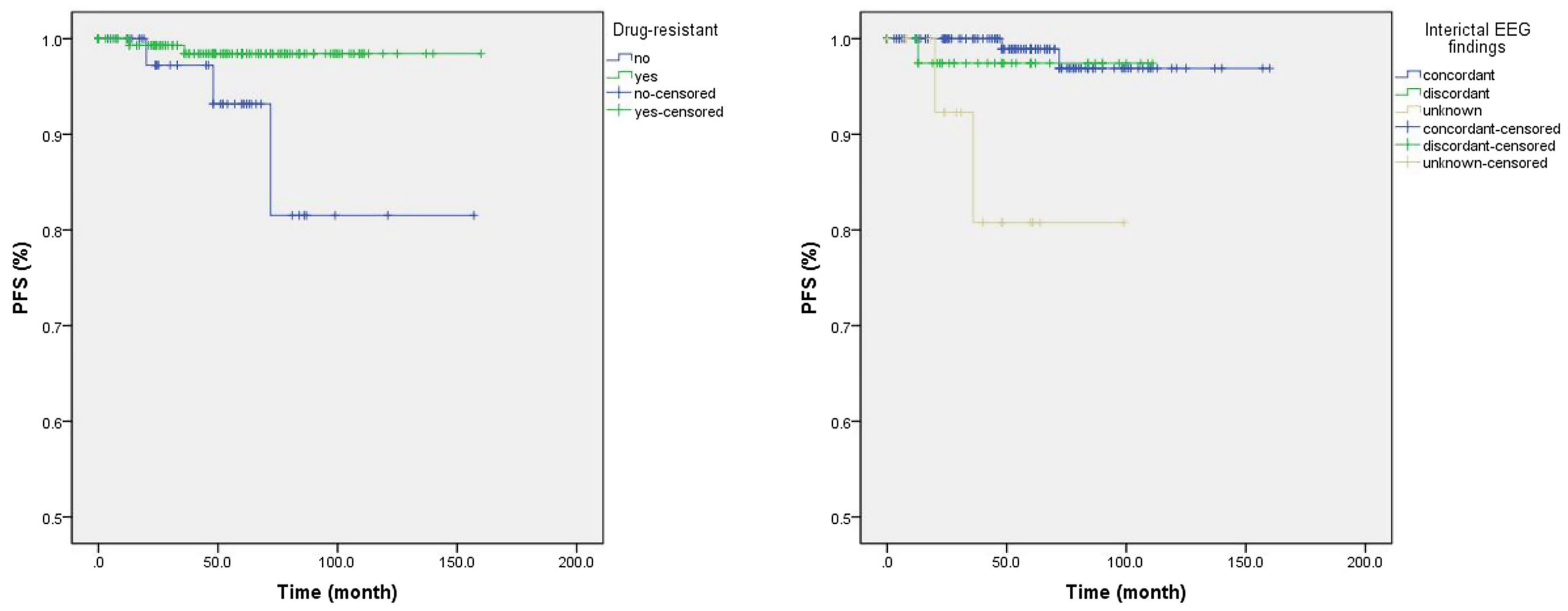
The Kaplan Meier curves of significant clinical factors in univariate Cox regression analysis. Patients with drug-resistant epilepsy were with a lower rate of tumor recurrence than those without drug-resistant epilepsy (Left; Log Rank test: *χ*^2^ = 5.551, *p* = 0.018), as well as in patients with concordant interictal video electroencephalogram (EEG) findings when compared to those with unknown EEG findings (Right; Log Rank test: *χ*^2^ = 11.76, *p* = 0.003).

**Figure 2:**
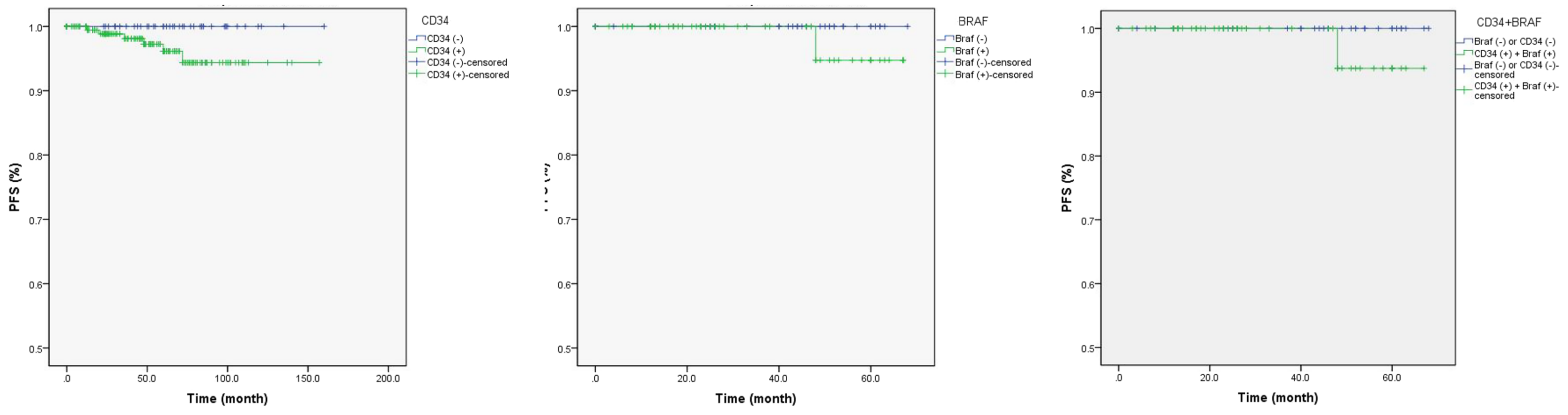
The Kaplan Meier curves of CD34 expression and/or BRAF^V600E^ mutation in gangliogliomas with epilepsy. CD34 expression (left; Log Rank test: *χ*^2^ = 0.832, *p* = 0.362) or BRAF^V600E^ mutation (middle; Log Rank test: *χ*^2^ = 0.824, *p* = 0.364) in gangliogliomas, or both (right; Log Rank test: *χ*^2^ = 0.938, *p* = 0.333), were not associated with tumor recurrence.

Univariate Cox regression analysis found drug-resistant epilepsy (HR = 0.15, *p* = 0.040) and concordant interictal EEG findings (unknown vs. concordant; HR = 14.75, *p* = 0.009) were associated with longer PFS (Table 4; Figure 1), but only the concordance of interictal EEG findings (unknown vs. concordant) was with significance in the multivariate Cox regression analysis. In particular, when compared the Kaplan Meier curves between groups [tumor with CD34 (+) vs. CD34 (−)] or groups [tumor with BRAF (+) vs. BRAF (−)], no difference was found in patients with detection of CD34 expression (*χ*^2^ = 0.832, *p* = 0.362) or in patients with detection BRAF^V600E^ mutation (*χ*^2^ = 0.824, *p* = 0.364), neither in patients with detection of both CD34 expression and BRAF^V600E^ mutation (*χ*^2^ = 0.938, *p* = 0.333; Figure 2).

**Table 4:** Univariate cox regression analysis of the associations of clinical factors with tumor progression-free survival.

*Variable*	*B*	HR (95% CI)	*p* value
CD34 expression	3.25	25.83	0.562
BRAF^V600E^ mutation	3.40	54.56	0.628
Patient gender (female vs. male)	−0.93	0.40 (0.04–3.54)	0.407
Patient population (adult vs. children)	1.18	3.24 (0.36–29.08)	0.293
Age at surgery, in years	0.06	1.06 (0.99–1.13)	0.117
Age of seizure onset, in years	0.06	1.06 (0.99–1.13)	0.085
Duration of epilepsy, in months	−0.01	1.0 (0.99–1.01)	0.744
Drug-resistant epilepsy	−1.88	0.15 (0.03–0.92)	0.040^c^
Seizure type (generalized vs. focal)	−0.69	0.51 (0.06–4.50)	0.538
Seizure aura	−1.56	0.21 (0.02–1.88)	0.163
History of GTCS	−1.84	0.16 (0.02–1.42)	0.100
History of SE	−3.03	0.05	0.828
Seizure frequency (monthly vs. non-monthly)	−0.01	1.0 (0.55–1.79)	0.990
Interictal EEG findings (discordant vs. concordant)	0.63	1.88 (0.17–20.81)	0.605
Interictal EEG findings (unknown vs. concordant)^a^	2.69	14.75 (1.96–111.1)	0.009^c^
Ictal EEG findings (discordant vs. concordant)	0.18	1.19 (0.11–13.17)	0.885
Ictal EEG findings (unknown vs. concordant)^a^	0.77	2.16 (0.30–15.39)	0.443
Tumor type (GG vs. GG-nos)	−3.09	0.05	0.711
Tumor size, in millimeter	0.03	1.03 (0.91–1.17)	0.625
Tumor side (left vs. right)	−0.33	0.72 (0.12–4.32)	0.720
Temporal invasion (temporal vs. non-temporal)	0.34	1.41 (0.16–12.59)	0.761
Medial temporal tumor	1.24	3.46 (0.58–20.70)	0.174
Tumor calcification	0.34	1.41 (0.23–8.46)	0.708
Tumor encystation	0.47	1.60 (0.18–14.46)	0.677
Ki67 index (2–5% vs. ≤1%)^b^	1.48	4.41 (0.73–26.67)	0.106
Tumor-associated FCD	−0.88	0.42 (0.04–3.79)	0.435
Concomitant HS	−3.11	0.05	0.697
Resection extent (extensive vs. simple)	0.45	1.57 (0.53–4.72)	0.417
New neurological deficit	−0.12	0.88 (0.16–4.87)	0.888
Hospitalization time, in days	−0.07	0.93 (0.83–1.05)	0.228
Follow-up time, in months	0.02	1.11 (0.99–1.14)	0.432

GTCS, generalized tonic–clonic seizure; SE, status epilepticus; EEG, electroencephalogram; GG, ganglioglioma; GG-nos, ganglioglioma, not otherwise specified; FCD, focal cortical dysplasia; HS, hippocampus sclerosis; HR, hazard ratio; CI, confidence interval. ^a^Patients with unknown results in lateral concordant EEG findings of interictal epileptiform discharges and of ictal seizure rhythms were those with no interictal epileptiform discharges or normal EEG findings and those with no ictus during video EEG monitoring. ^b^Including 2 cases with ki67 index recorded at 8 and 12%, respectively. ^c^*p* < 0.05, with significance.

## Discussion

The molecular markers of CD34 and BRAF^V600E^ mutation are frequently met in GG (Blümcke et al., 1999; Deb et al., 2006; Schindler et al., 2011; Xing et al., 2021). Although a few of pediatric gliomas share these molecular features with GG (Blümcke et al., 1999; Reifenberger et al., 2003; Giulioni et al., 2019), CD34 expression and BRAF^V600E^ mutation, as an adjunct diagnostic marker, are routinely screened for the diagnosis of GG (Blümcke et al., 1999; Schindler et al., 2011; Xing et al., 2021).

### The CD34 expression and BRAF^V600E^ mutation in gangliogliomas

As a molecular marker of progenitor cells, CD34 often expresses in low-grade or developmental brain tumors, which are usually accompanied by chronic epilepsy, such as the so-called “low-grade epilepsy-associated neuroepithelial tumors (LEAT),” but none of normal adult or developing human brain and tumors without epilepsy are CD34 positive (Blümcke et al., 1999; Deb et al., 2006; Chappé et al., 2013; Sidney et al., 2014; Giulioni et al., 2019). The GG are the most common tumor type in patients with epilepsy and also frequently reported with CD34 expression, ranging from 60 to 90% (Deb et al., 2006; Giulioni et al., 2019). In our study, we reviewed the CD34 expression in GG with epilepsy and found 88.5% of tumors were CD34 positive, which was line with the previous studies (Deb et al., 2006; Chappé et al., 2013; Giulioni et al., 2019).

The BRAF^V600E^ mutations that were primarily found in melanomas also occur in brain tumors, which, similar to CD34, mainly affect low-grade glial or glioneuronal tumors, such as GG, DNT, and PA, as well as pediatric PXA and diffuse astrocytoma (Schindler et al., 2011; Drosten and Barbacid, 2020; Xing et al., 2021). The mutation of BRAF^V600E^ in GG was reported ranging from 20 to 60% (Schindler et al., 2011; Prabowo et al., 2014; Vornetti et al., 2017; Xing et al., 2021). In present study, tumors with BRAF^V600E^ mutation were detected by IHC in 60.7% (54/89) of GG, including 61.4% (51/83) of GG and 50% (3/6) of mixed GG (not specified), but no difference of BRAF^V600E^ mutation was found between GG and mixed GG (*p* = 0.903).

### The clinicopathological features of CD34 expression

The relationship between clinicopathology and CD34 expression in GG has been studied in some studies, but the results were always inconsistent or in different types of tumors (Blümcke et al., 1999; Deb et al., 2006; Vornetti et al., 2017; Giulioni et al., 2019).

With respect to demographic features, for example, Blümcke et al. (1999) found patients with brain tumors with epilepsy and CD34 expression had younger age at seizure onset or at surgery and Vornetti et al. (2017) found CD34 expression in LEAT was significantly associated with a longer duration of epilepsy, which was similarly reported by Giulioni et al. (2019) who also reported that CD34 expression in LEAT appeared to be significantly related to older age at surgery, higher AED intake, and female sex by univariate analysis. In present study, we only found GG with CD34 expression occurred more in patients with older age (*p* = 0.017) by univariate analysis.

The tumor or pathological characteristics were less reported for GG with CD34 expression, except that Lisievici et al. (2021) found CD34 expression in GG was more in temporal lobe. In our study, we found GG with CD34 expression were less occurring in medial temporal sites than other sites (OR = 0.37; *p* = 0.030).

Although CD34 expression tends to occur in lesions with epilepsy, the seizure semiology or EEG finding is seldom reported to be related to GG with CD34 expression (Giulioni et al., 2019; Lisievici et al., 2021). In present study, however, we found patients with CD34 expression in GG had more tendency of experiencing seizure aura (OR = 2.94, *p* = 0.025) and concordant ictal EEG findings (discordant vs. concordant; OR = 0.34, *p* = 0.045) than those without CD34 expression.

### The clinicopathological features of BRAF^V600E^ mutation

Although the BRAF^V600E^ somatic mutation in neuronal linage cells (or glial lineage cells) was proved to play a key role in epileptogenic properties (or tumorigenic properties) of GG (Koh et al., 2018), the relationships between clinicopathological features and BRAF^V600E^ mutations in GG were not well studied or with less data (Dahiya et al., 2013; Koelsche et al., 2013; Prabowo et al., 2014; Vornetti et al., 2017; Zhang et al., 2017; Xing et al., 2021).

Several demographic features were reported to be related to GG or brain tumor with BRAF^V600E^ mutation, including younger age at surgery for GG (*p* = 0.005; Koelsche et al., 2013), younger age of seizure onset for epilepsy-associated brain tumors (*p* = 0.020; Xing et al., 2021), and female patients for glioneuronal tumors (GNT) with epilepsy (*p* = 0.022; Zhang et al., 2017). However, Schindler et al. (2011) noted no significant differences of patient age at surgery for GG with BRAF^V600E^ mutation; Zhang et al. (2017) reported no significant correlation between the BRAF status in GNT and age at surgery, as well as age of seizure onset and duration of epilepsy; and Xing et al. (2021) also did not find brain tumors with epilepsy and BRAF^V600E^ mutation were associated with gender and duration of epilepsy. In present study, we did not find any associations of BRAF^V600E^ mutation in GG with age of seizure onset, duration of epilepsy and patient gender, except for the statistic tendency (0.05 < *p* < 0.1) in older age at surgery (*p* = 0.064).

For tumor or pathological characteristics, Schindler et al. (2011) found GG with BRAF^V600E^ mutation were more in temporal lobe. Prabowo et al. (2014) found in both GG and DNT, the presence of BRAF^V600E^ mutation was significantly associated with the expression of CD34. Vornetti et al. (2017) found BRAF mutation in LEAT was predominant in right-sided lesions. However, Koelsche et al. (2013) found CD34 was not differentially expressed in BRAF wild-type and-mutated tumors of GG, and Xing et al. (2021) found there was no statistical difference between BRAF^V600E^ mutations and wild type for tumor site. Also, we did not find GG with BRAF^V600E^ mutation were associated with CD34 expression, tumor locations, calcification or encystation, et al.

With respect to seizure semiology or EEG findings, BRAF^V600E^-mutated LEAT (Vornetti et al., 2017), as well as GNT (Zhang et al., 2017), were reported to be with more seizure types. However, we did not find GG with BRAF^V600E^ mutation were associated seizure semiology, except that the statistic tendency existed in concordance of EEG findings was different (discordant vs. concordant; *p* = 0.078).

### Seizure outcomes and tumor recurrence

**Figure 3:**
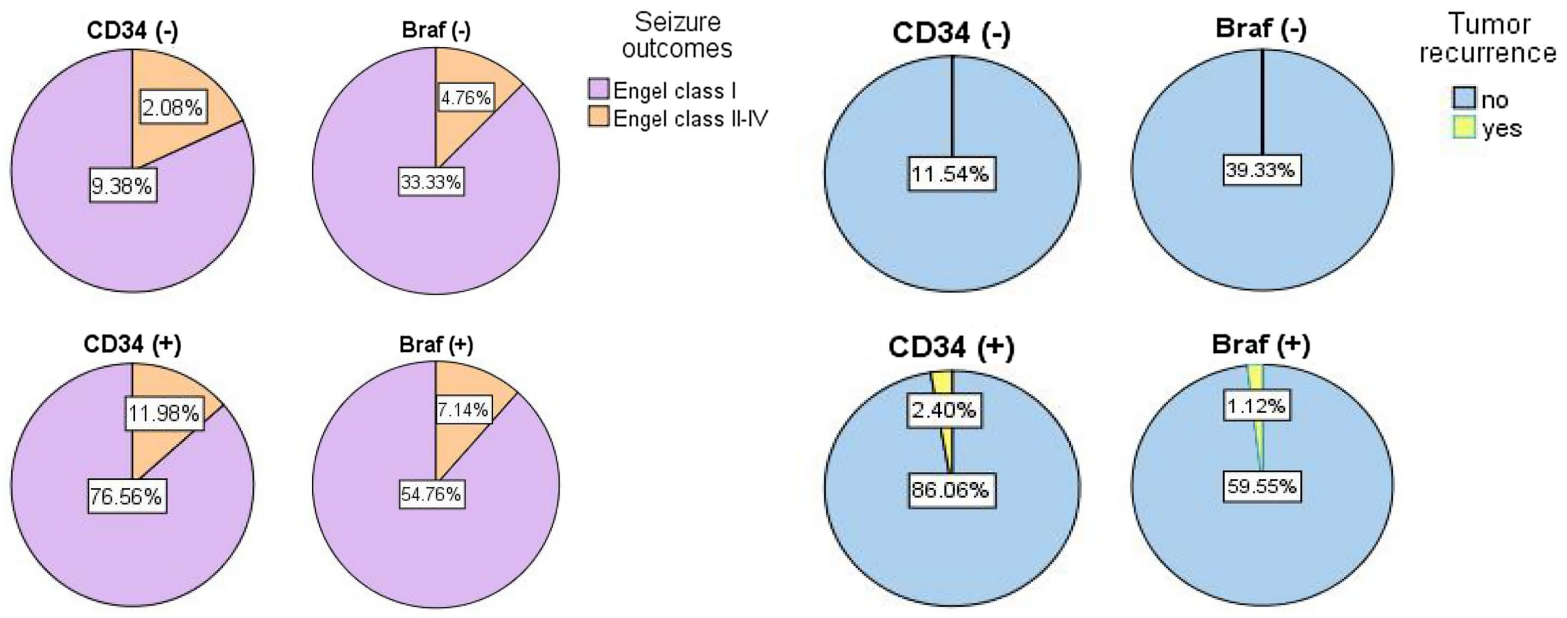
The pie graphs showing the percentages of prognostic results in seizure outcomes (left) and tumor recurrence (right) based on differential diagnosis of gangliogliomas with CD34 expression or with BRAF^V600E^ mutation.

The correlations between CD34 expression or BRAF^V600E^ mutation in GG and postoperative seizure outcomes have been evaluated in previous studies (Prabowo et al., 2014; Shen et al., 2017; Wang et al., 2022), but they were always with negative results (Vornetti et al., 2017; Zhang et al., 2017; Xing et al., 2021). For example, Wang et al. (2022) found 9 patients with GG had postoperative seizure recurrence, and 8 of them were immunoreactive for CD34, and Prabowo et al. (2014) found the expression of BRAF^V600E^ in GNT was associated with a worse postoperative seizure outcome. However, Vornetti et al. (2017) did not find LEAT with BRAF^V600E^ mutation or CD34 expression were associated with seizure outcomes. Zhang et al. (2017) did not find any significant correlations between the BRAF status in GNT and postoperative seizure freedom. Also, Xing et al. (2021) reported there was no statistical difference of epilepsy-associated brain tumors between BRAF^V600E^ mutations and wild type in Engel outcome comparison. Similarly, we defined no differences between CD34 expression (*p* = 0.807) or BRAF^V600E^ mutation (*p* = 0.937) in GG and postoperative seizure outcomes (Figure 3).

The GG are benign, slow-growing tumors, and patients with GG rarely experience tumor progression or recurrence, although 5% (or less) of GG with anaplasia or malignant progression were reported in previous studies (Zaky et al., 2018; Slegers and Blumcke, 2020). During the whole follow-up period of 54 months (ranging from 6 months to160 months) in our study, 5 patients had tumor recurrence and 2 cases had malignant progression (Figure 3), with the accumulated 10-year tumor PFS reaching 96%. Through univariate Cox regression analysis, we found drug-resistant epilepsy (HR = 0.15) and concordant interictal EEG findings (unknown vs. concordant; HR = 14.75) were associated with longer PFS, but only the concordance of interictal EEG findings (*p* = 0.009) was significant in the multivariate Cox regression analysis. In particular, when compared the Kaplan Meier curves between two groups [tumor with CD34 (+) vs. CD34 (−)] or groups [tumor with BRAF (+) vs. BRAF (−)], no difference was found in patients with detection of CD34 expression or BRAF^V600E^ mutation.

The relationship of CD34 expression or BRAF^V600E^ mutation in GG with tumor survival (PFS or overall survival) have been studied, previously (Chappé et al., 2013; Dahiya et al., 2013; Chen et al., 2017; Zaky et al., 2018; Lisievici et al., 2021). Although some of studies reported the significant correlation of CD34 expression or BRAF^V600E^ mutation in GG with tumor recurrence or progression (Lisievici et al., 2021), the extent of the surgical resection (or tumor location), instead of CD34 expression and BRAF^V600E^ mutation, may play an important role of the tumor prognosis of low-grade GG (Chappé et al., 2013; Dahiya et al., 2013; Zaky et al., 2018; Wang et al., 2022). However, when analyzing the association of tumor recurrence with resection extent or tumor locations, we did not find any statistic differences in resection extent (*p* = 0.417) and tumor locations (temporal vs. non-temporal, *p* = 0.761; or medial temporal vs. extra temporal, *p* = 0.174), which may be partly attributed to the high rate of complete tumor resection (99%) in our surgical cohort.

### Limitations

The evidence from our study with GG cohort may compromise its retrospective nature, as well as its inhomogeneity, such as in patient gender or age and tumor locations. However, we did not find any significant impacts of different patient gender (female vs. male), age population (children vs. adults) and tumor locations (temporal vs. nontemporal) on either CD34 expression (see Table 1) or BRAF mutation (see Table 3), or even surgical prognosis (see Table 4), in GG with epilepsy in our cohort. In addition, the limited follow-up time for patients with low-grade GG might weaken the evidence of our study when evaluating tumor recurrence or progression after surgery. Even so, our results could partly complement the undefined domains of the clinicopathological features of molecular alterations (CD34 and BRAF mutation) in GG with epilepsy, as well as the long-term surgical outcomes.

## Conclusion

CD34 expression and BRAF^V600E^ mutation are closely associated with GG in patients with epilepsy, which may also partly influence the distribution of clinical and pathological features in patients with GG. However, CD34 expression or BRAF^V600E^ mutation in GG may not impact the surgical prognosis of seizure outcome, as well as tumor PFS, if complete tumor resection could be achieved.

## Data availability statement

The raw data supporting the conclusions of this article will be made available by the authors, without undue reservation.

## Ethics statement

The studies involving human participants were reviewed and approved by Research Ethics Committee, Sanbo Brain Hospital. Written informed consent for participation was not provided by the participants’ legal guardians/next of kin because: The study was retrospective and does not contain individual clinical data, and thus informed consent was not required.

## Author contributions

M-GX and G-ML had the idea for the article. M-GX, JQ, and G-ML performed the data collection or analysis. M-GX, X-FW, and G-ML drafted and critically revised the work. All authors contributed to the article, read and approved the submitted version.

## Funding

This work was supported by the Open Cooperation Program of Chinese Institute for Brain Research, Beijing (2020-NKX-XM-02) and Special Scientific Research Project for Capital Health Development (2022–1-8011).

## Conflict of interest

The authors declare that the research was conducted in the absence of any commercial or financial relationships that could be construed as a potential conflict of interest.

## Publisher’s note

All claims expressed in this article are solely those of the authors and do not necessarily represent those of their affiliated organizations, or those of the publisher, the editors and the reviewers. Any product that may be evaluated in this article, or claim that may be made by its manufacturer, is not guaranteed or endorsed by the publisher.
